# We have **to** turn a transatlantic liner—Leaders' perspectives on creating a meaningful life for older persons receiving municipal care

**DOI:** 10.1002/nop2.1932

**Published:** 2023-07-05

**Authors:** Annica Kihlgren, Inger James, Margaretha Norell Pejner

**Affiliations:** ^1^ School of Health Sciences Örebro University Örebro Sweden; ^2^ Research Environment: Older People's Health and Living Condition Örebro University Örebro Sweden; ^3^ Faculty of Health, Science, and Technology Karlstad University Karlstad Sweden; ^4^ Department of Home Care Halmstad Municipality Orebro Sweden

**Keywords:** action research, elderly care, implementation, leaders perspective, meaningful daily life

## Abstract

**Aims:**

To describe leaders’ perspectives on what is important to create a meaningful daily life for older persons receiving municipal elderly care.

**Design:**

This study combined the Participatory Appreciative Action Reflection approach and qualitative methods.

**Methods:**

Focus Group Discussions were performed with eighty leaders that was analysed with qualitative content analysis.

**Results:**

One overall theme emerged “We have to turn a transatlantic liner”. The leaders proposed a need to change from an institutional care to a more person‐centered care approach on all levels of the healthcare system. This meant that everyone in the organization needed to think outside the box and find new ways to provide care to older persons. They needed to hire the right persons with the right values and knowledge. The leaders would need to provide for and support staff empowerment. There was also a need to see the older person and their relatives as co‐participants.

No Patient or Public Contribution.

## 1 INTRODUCTION

This article focuses on leaders' perspectives on a meaningful daily life for older persons in need of municipal health care and is part of a larger action research project (James et al., 2015) initiated as a result of reported shortcomings in Swedish elderly care. What constitutes a meaningful daily life for older persons, from the perspective of the older person, the relatives and the nurse assistants, has been reported (James, Blomberg, et al., 2014; James, Fredriksson, et al., 2014; Kihlgren et al., 2014; Windahl et al., 2014). However, there is a lack of knowledge from the leader's perspective on what constitutes a meaningful daily life for older persons and how to lead the work developing that in elderly care.

In Sweden, shortcomings in elderly care have been reported for a long time (Coronakommissionen, 2020; Ministry of Health and Social Affairs, 2010). The shortcomings are related to difficulties for the older person due to poor staff continuity or how to influence at what time(s) the staff members are to arrive (Socialstyrelsen, 2018). It appears that older couples in need of health care experience poor support and that support is more adapted to the organization than their individual needs (Norell Pejner & Brobeck, 2019). Fjordside and Morville (2016) show in a literature review is something that will counteract the autonomy and health of the older persons. To improve and promote the daily living situation of the older persons, the Swedish government introduced national guidelines to deal with the shortcomings and established core values and guarantees of dignity formulated at the local level (Socialstyrelsen, 2012). The guidelines highlight the need for dignity, well‐being and a perceived meaningful daily life for older persons in need of health care. The responsibility for the development and implementation of the guidelines was delegated to the municipalities. When guidelines are to be implemented, the leaders play a crucial role since the development of the quality of the care, the work environment and the methods used all fall within the scope of their duties (Ericsson & Augustinsson, 2015; Solbakken et al., 2019).

In terms of leadership in the context of elderly care it has been stated that leadership is getting increasingly important for setting the standards of the care (Backman et al., 2021). Within Swedish health care, leadership is implemented on various levels: the general level, the middle manager level and the first‐line manager level in homecare units and nursing homes. Regardless of the level of the leadership, the leaders do not work directly with patient care (Ericsson & Augustinsson, 2015). Their work consists of administrative duties such as the hiring and scheduling of the staff and keeping the economy in balance (Solbakken et al., 2019). In municipal home care of older persons, it is also important for the leaders to consider the relatives when making different decisions since they are involved in the care (Solbakken et al., 2019). Decisions need to be made regarding at what time(s) the care is to be provided, how the care should be designed to maintain safety and what is in the best interest of the older person (Jonasson, Sandman & Bremer, 2019). Relatives can have knowledge regarding the older persons' values and preferences, but since municipalities do not have infinite resources adaptations have to be made that can lead to ethical issues. There are also studies reporting job strain and work stress in elderly care among staff (Backman et al., 2018; Costello et al., 2019), stress of conscience (Åhlin et al., 2013; Backman et al., 2021) and significant problems retaining competent and stable workforce in elderly care, due to low job satisfaction (Edvardsson et al., 2011). How a leader responds and relates to their staff is reflected in the staff member's' attitudes and approaches to the older persons (Sellgren et al., 2008).

Leadership is a multifaceted concept; it is a process that influences and creates opportunities as well as facilitates carrying out tasks or problem‐solving in the complex organizational cultures in elderly care. They have an increasing number of staff members that they are responsible for. The staff members often have inadequate skills, which can contribute the inability to achieve the national and local goals in health care. Altogether, leaders play an essential role in elderly care when guidelines are to be introduced. Leadership is seen to be a key issue in how the context of clinical practice is shaped (Ericsson & Augustinsson, 2015; Solbakken et al., 2019). The leadership style and their attitudes will influence how they lead the implementation phase of the guidelines. This in turn affects the staff members' ability to implement the care changes that will create an environment and an everyday life that older persons can perceive as meaningful. To succeed with the implementation of the guidelines, there is the need for a shared vision on what constitutes a meaningful daily life for older persons in municipal care. However, there is a lack of knowledge from the leader's perspective on what constitutes a meaningful life and how it can be created for older persons receiving municipal care. The aim of the present study was therefore to describe the leaders' perspectives on what is important to create a meaningful daily life for older persons receiving municipal elderly care.

## 2 METHOD

This study combined the Participatory Appreciative Action Reflection (PAAR) approach and qualitative methods (Ghaye et al., 2008). Eighty leaders participated in Focus Group Discussions (FGDs) held on two different occasions. This study was part of a larger action research project, with a focus on what constitutes a meaningful daily life for older persons living in ordinary housing with home care or in nursing homes. The larger project aimed to seek obstacles, opportunities and solutions for the development of a meaningful daily life from the older persons' perspective by working together with the older persons, their relatives, staff and leaders. The larger project also aimed to use these data to formulate core values and local guarantees of dignity in municipal care which would be implemented in the care of older persons (redacted) and evaluated (redacted). The project is a result of the Swedish Government's decision that elderly care should lay on core values and local guarantees of dignity in all municipalities. This municipality chose a bottom‐up perspective and decided to develop core values and local guarantees of dignity from the older person's perspective. In total, 386 persons participated in the larger PAAR project. In all, we worked with stakeholders, including older persons, relatives, organizations for retired persons, nurse assistants, registered nurses, occupational therapists, managers and politicians.

### 2.1 Recruitment of respondents

This project was conducted in one municipality in the middle of Sweden. At the time of the study, the municipality had 2700 co‐workers and 5000 older persons enrolled in health care and social services. A convenience sample was used by inviting all leaders (*n* = 93) from the three levels of elderly care in this municipality to participate. Thirteen declined due to high workloads or were off duty, and the 80 who accepted were included in the study. There were two from the general level, eight from the middle manager level and 70 from the first‐line manager level. Thirteen declined participation due to the lack of time. Among the leaders at the general level was one who was responsible for the development of all care in the municipality and one who was the head of operations. The eight middle managers headed the eight different healthcare sectors in the municipality. Among the first‐line managers were two who were responsible for two private nursing homes, 34 who were responsible for municipal nursing homes and 28 who were responsible for municipal home care units. Six of the first‐line managers were responsible for meal deliveries, day care and cleaning. Their educational backgrounds varied. The leaders at the general level were nurses. The education among the other leaders varied between nursing and social work, with a preponderance in social work. There were 73 women and seven men, with a mean age of 49.4 years (range 29–66). They had worked as leaders between one and 18 years (mean 14 years).

### 2.2 Data collection

Ten researchers from one research milieu with focus on elderly research, one associated prof and seven PhD lectures and two experienced teachers, from one University were involved in performing the FGDs according to standard FGD procedures (Kvale & Brinkmann, 2009; Thorne, 2008). The manager who was responsible for the staff in the municipality invited all leaders and organized the time and place for the FGDs. The 80 leaders who agreed to participate after they had signed the written consent were divided into 10 mixed groups with six to 11 participants each. The moderators who led the FGDs consisted of one of the researchers together with a person with experience from leading group discussions in the municipality who took notes and assisted in asking clarifying questions. The FGD were held in a conference centre and the leaders participated in at least one session. If they were unable to participate in a second session, they read a transcript of the first session and submitted written or oral reflections and comments that would be presented by proxy at the second session. In line with our bottom‐up perspective within action research (Bradbury Huang, 2010, 2012) and in cooperation with stakeholders, we only asked one open‐ended question: *What is a meaningful daily life for older persons*. Follow‐up questions were raised to gain reflections on what factors could influence a meaningful daily life for older persons and how a meaningful daily life could be created. During these conversations, the researchers and the leaders reflected on and sought various ways of understanding the opportunities, obstacles and solutions for a meaningful daily life. The first FDGs that lasted between 60 and 90 min were digitally recorded and transcribed verbatim by an experienced secretary. Before the second FGDs, the leaders read the transcriptions and together with the researcher provided their analyses and reflections on the content to get a deeper understanding of what had been said. The second sessions that lasted between 40 and 60 min were also recorded and transcribed verbatim by an experienced research secretary.

### 2.3 Data analysis

To analyse the data, a qualitative content analysis was performed by the authors in a number of steps. The first author read through the transcripts of the FGDs several times to acquire a general sense of the whole. Meaning units related to the same central meaning and relevant to the aim of the study were identified and discussed with all authors. The meaning units were then condensed into a description close to the text to grasp the manifest content and coded. The manifest content of those codes was sorted based on their similarities and differences into either obstacles or opportunities for a meaningful daily life for the older persons. The underlying meaning in the condensed meaning units and codes was then interpreted to determine the latent content (Graneheim & Lundman, 2004), see Table 1.

**TABLE 1:** Overview of categories and the theme developed from the interviews.

Meaning units	Condensed meaning units	Code	Themes	Overall theme
‘Yes, it's what we usually say – routines are stronger than what they almost should be. All of the routines are very strong – that you should do it exactly as … I think I, people see it that way … This is how we've done it every Wednesday and so shall it be. But it is beginning to soften up. But the routines are so strong. We need to work more with that, find new ways’.	We must find new ways	New ways of thinking	Thinking outside the box	We have to turn a transatlantic liner
‘Being present … that you are in the moment … are here. Some staff just have it … they see what is important and can make something of it’	Some staff have own resources and take the initiative	Some staff are better suited than others	Hiring the right person with the right values and knowledge	
‘I think a person must help the staff to take inventory, to check what the person that has just come here or that I have in front of me – what does that person want? And what can I offer? Because if you ask; what do you want to do? Yes, in relation to what?’	Somebody must help the staff to obtain structure in their work	To provide structure	I as a leader have to provide for and support staff empowerment	
‘It is important how you work with the relatives…They are also individuals, you can't meet them as a group…We have to be more flexible here. Having different kinds of meetings, find new ways… study circles can be one way’	Relatives cannot be seen as a group. We must meet them in their situation and learning from them	See relatives as a resource	See older persons and their relatives as co‐participants	

The codes were then sorted into subthemes and four themes: *Thinking outside the box, Hiring the right person with the right values and knowledge, I as a leader have to provide for and support staff empowerment, and See older persons and their relatives as co‐workers*. After summarizing the latent content in the themes, an overall theme emerged: ‘*We have to turn a transatlantic liner*’. During the analysis, the first author scrutinized and repeatedly discussed the results with the authors and the researchers within the research environment to ensure the trustworthiness of the data analysis and the best form for presentation. The quotations that were translated from Swedish to English by an experienced translator are presented below to illustrate the findings.

### 2.4 Ethics approval and consent to participate

The research project was approved by an Ethical Review Board (Redacted). Oral and written information was given to the participants, and the 80 leaders who agreed to participate signed the written consent for participation.

## 3 FINDINGS

The leaders believed there would be much work on several levels ahead of them before a meaningful daily life could be created for older persons in municipal care. They would need to work to get everyone in the entire organization to think outside the box and find new ways to provide the care to the older persons. The right persons with the right values and knowledge would need to be hired and the leaders would need to provide for and support staff empowerment. There was also the need to have everyone see the older persons and their relatives as co‐workers. They compared this massive amount of work on all levels to be like turning a transatlantic liner as the overall theme shows. To turn their transatlantic liner, the leaders proposed a need to change from an institutional care approach to a more person‐centred care approach on all levels of the municipal elderly care system.

### 3.1 Thinking outside the box

One opportunity and necessity for creating a meaningful everyday life was to use this new starting point to break with the ‘old ways’ of thinking. It was important to think outside the box to find new ways of working and let the older persons voice be the starting point in the planning and deliverance of all care. The leaders felt it was necessary to increase the older persons' participation in the care process. They considered it necessary to find new ways since meaningfulness can often be found in ‘that little extra’. To succeed with that, there was a need to shift the power of the care to the older person.

What is important is … that you don't sit there before and plan in groups with the personnel and co‐workers and then meet the older person … it is the older person's points of view that you should begin with … we need to shift the power to the older person.

The older persons were looked upon as a collective entity instead of as individuals. It was therefore important to open up new ways of thinking to find and create meaningfulness for the individual. To create a meaningful daily life, the care should be tailored to the needs of the individual and flexibility is needed in those situations where it was possible to do the little extra that the old person considers meaningful. The leaders witnessed too much of this type of thinking of the older persons as a collective group throughout the entire organization and proposed more flexibility in the system to create meaningfulness.

We should be able to open up a new way of thinking – not always stuck in the same mind set. We are rather set in our ways. There must be a totally different flexibility here – you have to take it as it comes.

The leaders expressed that it was necessary with routines, but current routines did not allow for the power to be shifted to the older persons. Leaders felt there was a need to work with their staff members to help them think in a new way and be more ingenious and flexible.

They reflected over several ideas for changes that had come up during the years that could have helped create a meaningful daily life for older persons but felt that the ideas had not been given proper attention from the government officials. They realized that resources were limited, but when thinking outside the box new ideas such as different activities could be actualized. Often, there were several good ideas that could not be actualized due to a disparity between what they wanted to do and what they had resources for. To succeed, there was a need to break patterns. The leaders also reflected over how staff resources were needed if they were to find new ways to work within the existing limits.

Yes, it's what we usually say – routines are stronger than what they almost should be. All of the routines are very strong – that you should do it exactly as … I think I, people see it that way … This is how we've done it every Wednesday and so shall it be. But it is beginning to soften up. But the routines are so strong. We need to work more with that, find new ways.

They also saw a need for thinking outside the box to create staff continuity, because with the large staff turnover it was considered a large problem. All the leaders stated that staff continuity was essential if meaningful daily lives for the older persons were to be achieved. Staff continuity helps to create alliances and relationships that can lead to a sense of community that was considered by the older persons to be important for a meaningful daily life.

### 3.2 Hiring the right person with the right values and knowledge

The leaders reflected on the importance of hiring the right staff. The hiring process should be designed so that the basic values important to the organization corresponded with the new employees' values. Correct values meant being engaged in one's work, liking older persons and being driven by curiosity. They saw a collective need to re‐examine the routines for hiring new staff and to find new ways to hinder the hiring of staff with the ‘wrong’ values.

Yes, so just this fundamental part is so incredibly important, it is the kind of thing that I have had with me that I feel has become even more clear, how should we recruit. Change our recruiting based on our basic values. We have to find a solution for that?

The leaders concurred that staff members can be better suited for different things. Some of the staff are more task oriented and prefer to clean and wash while others would rather create meaningfulness through different activities that they feel the older person would enjoy. Since both types of staff were needed, it was the responsibility of the leaders to organize them so they could work in the best way. The leaders pointed out the importance of each staff member's ability to be sensitive to what they saw when meeting the older person and then trying to create activities based on that. Knowledge of the older person's life history that was written down in the patient records was a starting point and often a basis for individualized care.

Being present … that you are in the moment … are here. Some staff just have it … they see what is important and can make something of it.

The leaders often reflected in the interviews on the importance of having staff members that know what to do. It was also considered important that the staff focused on the opportunities instead of the obstacles when attempting to create meaningfulness.

And try not to see the hinder but look a little for the possibilities. Because actually there is nothing that isn't possible – although it can be done in different ways.

### 3.3 I as a leader have to provide for and support staff empowerment

The leaders reflected over their role in giving support to the staff. It was also considered important to reflect over ethical questions together with the staff and for the leaders to have the courage to ask difficult questions.

I also feel that we need to be better at daring to talk about the more ethical issues. We talk a lot about practical things like potato skins and walks but this with daring to ask those questions – such as, how a person feels and how they feel as a person and what desires and thoughts they have. It is a difficult area that we don't all manage or dare to talk about.

Leading and supporting the staff was a way to create meaningfulness for older persons. The leaders felt by working closely with their staff they could get to know them better. They considered it import to strengthen their staff's self‐confidence and ability to succeed in their work.

I think that you must give a push every Monday. We go around every morning. What are the plans for today? What have you all thought about this week? Then encourage them a whole bunch; Oh good, yes, do that.

As leaders, their roles were to support their staff in trying to find different possible ways that could create meaning in the older persons' daily lives and to create conditions for the staff so they could find the ‘little extra’ in the situation and do something about it. To accomplish that, they would need to help the staff ascertain the older person's own agenda and to work from it. They suggested different solutions such as working together with the staff using good examples and encouraging reflection as methods to learn new things and to change old behaviours.

### 3.4 See older persons and their relatives as co‐participants

Another way to create a meaningful life for older persons was to see them and their relatives as co‐participants. The leaders thought it was important to involve the older person and their relatives in the care to create more individualized care.

Who knows the most, is it us as caregivers or is it the elderly themselves or can it be their relatives, do we make use of the relatives' knowledge?

The leaders saw it as an opportunity for a meaningful life for the older person when the staff members had the courage and expertise to meet with the older person or their relatives in order to assess their desires and strengths and encourage them do what they could. The leaders commented on how some of the staff members find it difficult to listen to the relatives and to get them to cooperate.

I have many co‐workers and nursing assistants that have difficulties listening to relatives … we need to help them with that.

The leaders discussed the difficulties in getting the older person and their relatives to be co‐participants instead of acquiescing and adapting to the system. They felt that if the older persons and their relatives constantly adapted, they would never become involved in the care. The leaders also reflected over several new ways of helping the relatives to be more involved as they believed the relatives played an important role in the creation of meaningfulness in the older person's daily life.

It is important how you work with the relatives … They are also individuals, you can't meet them as a group … We have to be more flexible here. Having different kinds of meetings, find new ways … study circles can be one way.

## 4 DISCUSSION

The leaders in this study highlighted the need for a change in the culture of elderly care. They proposed leaving the institutional care approach for a more person‐centred care to create a meaningful daily life for older persons. The leaders felt that they had much work ahead of them on several levels, which was an undertaking as titanic and difficult as turning a transatlantic liner. The responsibility for implementing a meaningful daily life for older persons enrolled in municipal health care was delegated to the leaders in this study by the government. There are many models of leadership that work in different situations, but what is primarily needed here may be a shared vision. To succeed, what can be needed is to develop a vision and then put processes in place that constantly sell the vision to the co‐workers on all levels. McCormack and McCance (2017) suggest that leaders need to work with their teams to develop a shared vision of person‐centredness. Hersey and Blanchard (1997) stated that once a leader has clarified and shared the vision, they can focus on serving and being responsive to the needs of the people. They need to understand that their role as a leader is to remove barriers so staff members can achieve the vision (Mc Cormack & Dewing, 2010) of creating a meaningful daily life for older persons and implement the core values.

The leaders saw that successful implementation of a meaningful daily life for older persons would require everyone in the entire organization to think outside the box and try to find new ways. Having a shared vision could be one way to succeed. This is in line with Hersey and Blanchard (1997) who point out that when everyone supports the organizational vision a deliberate, focused culture that drives the desired outcome is created. It requires courage to do that and the ability to listen to the new things being said and to put previous values aside. This means that when a person is in a conversation with another, they need to be open and not limited by an unwavering idea of where the conversation should end (Gadamer, 2004).

McCormack and McCance (2017) stated that this shared vision incorporates the desired outcomes for person‐centred practice, which include satisfaction with care, feeling involved in care, having a feeling of well‐being and the existence of a therapeutic environment. This environment is described as one in which decision‐making is shared, staff relationships are collaborative, leadership is transformational and innovative practices are supported. The leaders also need to promote staff continuity, which contributes to the development of a relationship between the staff and older persons. With such a relationship they can get to know each other and develop a sense of trust, both of which are necessary for the development of a meaningful daily life. This is in line with other studies (James, Blomberg, et al., 2014; James, Fredriksson, et al., 2014; Kihlgren et al., 2014) and clinical experiences in the Swedish healthcare system that highlight the issue of staff continuity. Knowledge of an older person's life history, favourable conditions for the development of a therapeutic relationship and appropriate interventions are fundamental in person‐centred care (Kellett et al., 2010).

To succeed, it seems that common visions and values are required among all the employees and that the right people are hired (Mc Cormack & Dewing, 2010). The leaders considered the hiring of the right person important since having staff members who can be sensitive to older persons when interacting with them was vital for the creation of meaningful activities. James, Blomberg and Kihlgren et al. (2014) state in their study that emotional involvement helped nurse assistant to sense and read the older person to judge how the person was feeling. Based on the results, the authors concluded that nurse assistant used emotional involvement to develop meaningful daily lives for the older persons and give meaning to their work.

The leaders in this study saw the need to work closely with their co‐workers in order to provide for and support empowerment to their staff during the process. As leaders, they need to give the employees authority and the power to act. The employees need to have control over their decisions, which in turn gives them a positive psychosocial work environment. Shields and Norton (2006) state that a key principle of culture change is a person‐centred practice whereby those receiving health care and the staff become empowered and self‐determining decision‐makers. The managers need to empower the staff so they have power over their work, and thereby can in turn give empowerment to the older persons (Lundgren et al., 2016). The managers need to give the employees authority and the power to act, so they can have control over their decisions and positive psychosocial work environments.

To be successful in creating meaningfulness and person‐centred health care, the leaders saw the importance of involving the older person and their relatives in the care. An obstacle occurred when the staff members had difficulties listening to the relatives. By providing information and biographies of their older family members, relatives may experience satisfaction and empowerment, which could increase participation (Kellett et al., 2010). However, the leaders saw difficulties in getting the older person and their relatives to be involved as they often adapted to the care. Questions arise about why older persons seem so reticent and what consequences that will have for the leaders with the turning of their transatlantic liner. Older persons may prefer to comply rather than voice their preferences, which makes person‐centred care more difficult to achieve. When they conceal their needs, there is a risk that their care will become task oriented. The staff can also give support to the older persons and help them be true to themselves rather than simply adapting (James, Ardeman‐ Merten, et al., 2014). Person‐centred care should include a partnership between the elderly, close relatives and staff (McCormack & McCance, 2017).

David Edvardsson (2015) problematizes and contributes with a similar perspective. He believes cooperation with the person and close relatives comes from a personal approach built on social interpersonal interaction. This requires an interest in the person and their view on their own health. He refers to Martin Buber (1970) and believes that person‐centred care should rest on an I/You relationship where the emphasis is on the who rather than an I /It relationship where the tasks and actions are the focus. Person‐centred care is holistic, flexible, creative, personal and unique, and not standardized, reductionist and task oriented unless the person prefers to be treated that way (Edvardsson, 2015). To work using person‐centred care, you need commitment and compassion in your desire to help another person and ease their suffering. Additionally, support is needed for reflection so that you can understand both yourself and the context in which the care is taking place (Backman et al., 2018; Backman et al., 2020; van Lieshout et al., 2015). To make it possible for older persons to live true to themselves, their personality and identity; leadership is required that fosters creativity in employees so they can create person‐centred care and a meaningful daily life for older persons.

### 4.1 Methodological considerations

In action research, it is important that the participants take part in it as co‐researchers (Bradbury Huang, 2010). In the present study, the leaders can be seen as co‐researchers in the data collection process. They were able to change, delete or advise shortcomings as well as add to the data. The research was also associated with some limitations. All the leaders participated in the FGDs one time, but not all physically attended a second time. To accommodate for this, the researchers decided that if the leaders could not attend the second time, they could participate by proxy. They had to read a transcript of the first interview and submit their reflections and comments in writing or orally to a colleague for presentation during the second FGD. The leaders considered their participation in the research as something positive. By holding the FDGs on two occasions and with the method used during the FDGs, opportunities for reflection were enhanced resulting in a deepened knowledge of how to create a meaningful daily life for older persons receiving municipal care. This can be seen as a form of credibility (Adili et al., 2013), that is strength in the study. During the interpretation process, we attempted to maintain an open attitude (Dahlberg et al., 2008) and be open to the text. Due to that the stakeholders cooperated with the researcher it strengthened the conformability, that is trustworthiness. All the leaders represented the whole elderly care organization in one municipality, and it is reasonable to assume that there is transferability in the results to other elderly care organization in Sweden.

In conclusion, the leaders in this study highlighted the need to change to a more person‐centred approach to create a meaningful daily life for older persons. They compared it with striving of trying to turn a transatlantic liner. The responsibility to execute these changes was assigned to them by the government. Having a shared vision of the goal throughout the entire organization was considered necessary. Thinking outside the box and finding new ways to provide health care to older persons was needed. The individual's voice: that of the older person should be the starting point in the planning and deliverance of all care. New ways are needed to be found to create staff continuity. The hiring process should be reviewed and designed so that the basic values important to the organization corresponded with the new employee's values and knowledge. The leaders considered it important to provide empowerment and to strengthen their staff's self‐confidence so they could succeed in their work creating a meaningful life for older persons. There was also a need to see the older person and their relatives as co‐workers. To create a person‐centred healthcare approach, the leaders need to support their staff members so that they in turn can give support to the older persons. Common visions and values need to be developed on all levels in the care of older persons to make it possible.

## AUTHOR CONTRIBUTION

All authors have contributed significantly and are in agreement with the content of the manuscript.

## FUNDING INFORMATION

None.

## CONFLICT OF INTEREST STATEMENT

The authors report no conflict of interest.

## ETHICS STATEMENT

The research project was approved by the regional Ethical Review Board in Uppsala (Reg. No. 2011/009).

## Data Availability

Intervju data is avaliable in Swedish.
